# Development and internal validation of a machine-learning model for 3-year mortality after lung cancer resection: an online risk calculator and nomogram

**DOI:** 10.3389/fmed.2026.1815915

**Published:** 2026-09-25

**Authors:** Chen Liu, Yahui Wu, Jun Wang, Ke Lei, Yixiang Xing, Min Kang, Qihang Gao, Yun Shen

**Affiliations:** 1 Department of Pathology, Tongling People’s Hospital, Tongling, Anhui, China; 2 Tongling Academy of Medical Sciences, Tongling, Anhui, China; 3 Department of Pathology, Heping Hospital Affiliated to Changzhi Medical College, Changzhi, Shanxi, China; 4 Department of Respiratory and Critical Care Medicine, Tongling People’s Hospital, Tongling, Anhui, China

**Keywords:** feature selection, logistic regression, lung cancer, machine learning, mortality prediction, nomogram, web-based calculator

## Abstract

**Background:**

Lung cancer remains a leading cause of cancer-related mortality, and postoperative risk stratification may support individualized surveillance after pulmonary resection. This study aimed to develop and internally validate an exploratory prediction model for 3-year postoperative mortality using routinely available clinical, pathological, and laboratory variables, while explicitly addressing outcome ascertainment, model calibration, and the limitations of a single-center retrospective cohort.

**Methods:**

LASSO regression was used for feature selection, followed by a comparison of seven algorithms: Logistic Regression, Decision Tree, Random Forest, Support Vector Machine, Artificial Neural Network, XGBoost, and LightGBM. Model discrimination, calibration, and decision-curve performance were evaluated in a 7:3 internal hold-out split. The final model was selected primarily according to an internal-validation AUC, with calibration and classification metrics considered jointly. A web-based calculator and a nomogram were developed from the final model.

**Results:**

In the final outcome-ascertained analytic sample, 166 of 350 patients (47.43%) were classified as having died within 36 months and 184 (52.57%) were alive beyond 36 months. This proportion describes the event composition of the selected analytic cohort and should not be interpreted as a population-level postoperative mortality estimate. Six predictors were retained by LASSO: T stage, airway dissemination, pleural invasion, Ki-67 index, BMI, and direct bilirubin. Random Forest showed perfect apparent discrimination in the training set (AUC = 1.00) but decreased to 0.73 in validation. Logistic regression achieved the highest validation AUC (0.79; 95% CI, 0.70–0.87), with an accuracy of 0.75, an F1 score of 0.73, and a Brier score of 0.187, and was therefore selected as the final model.

**Conclusion:**

The final logistic regression model provided moderate internal discrimination and an interpretable framework for exploratory 3-year mortality risk estimation after lung cancer resection. The nomogram and web-based calculator facilitated model visualization, but the outcome-ascertained analytic cohort, potential event enrichment, and absence of external validation limited the interpretation of absolute risk and direct clinical application. Therefore, an independent multicenter and prospective validation with appropriate recalibration is required.

## Introduction

Lung cancer remains one of the most prevalent and deadly cancers worldwide. According to recent global statistics, lung cancer accounts for approximately 11.6% of all new cancer cases and 18.4% of cancer-related deaths, making it the leading cause of cancer mortality globally (1–3). The prognosis for lung cancer remains poor, with a 5-year survival rate for all stages of the disease hovering around 10%–20% (4). The economic burden of lung cancer is also staggering, with direct medical costs, including surgery, chemotherapy, and palliative care, exceeding billions of dollars globally every year (5, 6). These figures highlight the urgent need for improved diagnostic and prognostic tools to enhance early detection and treatment outcomes.

Lung cancer is often asymptomatic in its early stages, making early diagnosis and accurate prognostication crucial for improving patient outcomes. Survival is influenced by a variety of factors, including tumor stage, histological subtype, comorbid conditions, and treatment modalities (7–9). Despite significant advances in treatment, the prognosis for many patients remains poor. Therefore, there is a need for interpretable tools to estimate postoperative mortality risk in lung cancer patients, particularly for 3-year mortality risk assessment, which may support exploratory risk stratification and follow-up planning. In this study, we aimed to develop a machine learning–based model to estimate 3-year mortality risk in lung cancer patients using routinely available clinical, pathological, and laboratory data. Machine learning has demonstrated significant advantages in predictive modeling, providing the potential for higher accuracy and better generalization than traditional statistical methods (10). However, the translation of machine-learning models into clinical practice requires not only discrimination performance but also transparent reporting, interpretability, appropriate validation, and careful assessment of generalizability before clinical implementation (11, 12).

Moreover, existing studies on survival prediction for lung cancer patients still have several limitations, including heterogeneous study populations, limited interpretability, inconsistent validation strategies, and insufficient translation into practical tools for individualized risk estimation (11–13). Compared with previous studies, the present study focused on patients who underwent pulmonary resection and used routinely available demographic, pathological, and laboratory variables that can be obtained in standard clinical practice. In addition, we combined LASSO-based feature selection, multiple machine-learning algorithms, and an explicitly interpretable final regression model with graphical risk presentation to improve transparency and usability (14, 15). The intended clinical value of the model is exploratory postoperative risk stratification rather than direct treatment selection. Because the model was derived from a retrospective single-center cohort and evaluated only with an internal hold-out set, it should be considered a preliminary research tool pending independent external and prospective validation.

## Materials and methods

### Study design and population

This retrospective single-center study was conducted at Tongling People's Hospital, Anhui Province, China. Patients who underwent pulmonary resection for pathologically confirmed lung cancer between January 2020 and December 2022 were screened. The present analysis was restricted to patients for whom the 36-month postoperative vital status could be definitively ascertained from the available follow-up records. Patients who died within 36 months after surgery had an ascertainable event, whereas survivors were included only when survival beyond 36 months could be confirmed. Patients whose 36-month vital status remained indeterminate were not included in the analytic dataset. A total of 350 patients with definitive 36-month outcome status were available for analysis. Lung cancer diagnoses were based on histopathological examination and independently reviewed by two senior pathologists (16, 17). The participant-selection process is shown in Figure 1. Because death occurring within 36 months established the endpoint immediately, whereas a non-event required sufficient observation to confirm survival beyond 36 months, the final cohort should be regarded as an outcome-ascertained analytic sample rather than an unselected population-based surgical cohort. The historical screening log did not permit reliable reconstruction of the exact number of otherwise eligible patients excluded solely because their 36-month status remained unresolved; therefore, no unsupported exclusion count was retrospectively inferred.

**Figure 1:**
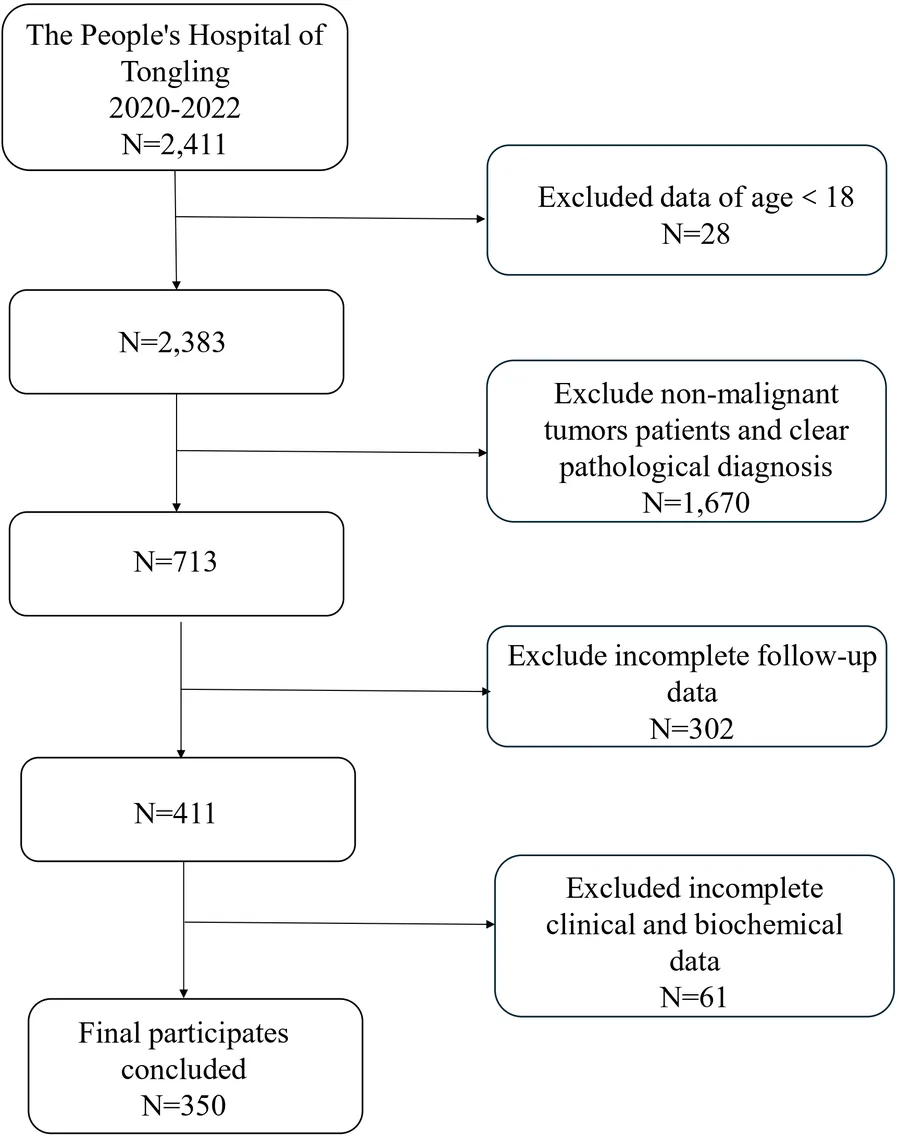
A flowchart of patient inclusion and exclusion. The flowchart depicts the construction of the outcome-ascertained 36-month analytic cohort. Patients entered the analysis only when postoperative vital status at 36 months could be definitively determined: death within 36 months established an event, whereas non-event status required confirmed survival beyond 36 months. Patients with indeterminate 36-month status were not included. The final analytic cohort comprised 350 patients.

### Outcome and predictors

The primary outcome was fixed 36-month postoperative mortality. Before reanalysis, the outcome field was audited against the coding dictionary and patient-level records: Outcome = 1 denoted death within 36 months after pulmonary resection and Outcome = 0 denoted survival beyond 36 months. Survival status was ascertained from postoperative telephone follow-up, outpatient records, and hospital medical records. Patients whose 36-month vital status could not be definitively ascertained were not included in the analytic cohort. Based on previous research (18, 19), candidate predictors included sex, age, histological type, differentiation grade, T stage, lymph-node metastasis, nerve invasion, vascular invasion, airway dissemination, pleural invasion, maximum tumor diameter, Ki-67 index, BMI, smoking and alcohol status, hypertension, diabetes, cardiovascular disease, family history, hemoglobin, carcinoembryonic antigen (CEA), neutrophils, total cholesterol, albumin, high-density lipoprotein cholesterol (HDL), low-density lipoprotein cholesterol (LDL), triglycerides, and direct bilirubin (20, 21). Predictor definitions and coding were finalized before model fitting to prevent outcome-label ambiguity.

### Data preprocessing

The outcome was analyzed as a fixed-time binary endpoint. The final analytic cohort contained only patients with a definitive 36-month status, and the locked coding used throughout model development was 1 = death within 36 months and 0 = alive beyond 36 months. The 350 patients were divided into a training set (*n* = 246) and an internal hold-out validation set (*n* = 104) at a 7:3 ratio. No patient in the internal validation set was used for variable selection or model fitting.

The data split and validation strategy followed standard principles for estimating generalization performance (22). Both subsets originated from the same institution; therefore, the hold-out subset was explicitly treated as internal rather than external validation. During initial data extraction, missing laboratory entries were identified and returned to the source medical records for verification and completion. The initial missing-data audit is shown in Figure 2. Statistical imputation was not used in the final locked analysis (23). The dataset used to generate the revised tables and figures contained complete values for the model variables. Predictor coding was applied consistently to the training and validation sets, and no information from the validation outcomes was used to alter feature definitions or model structure.

**Figure 2:**
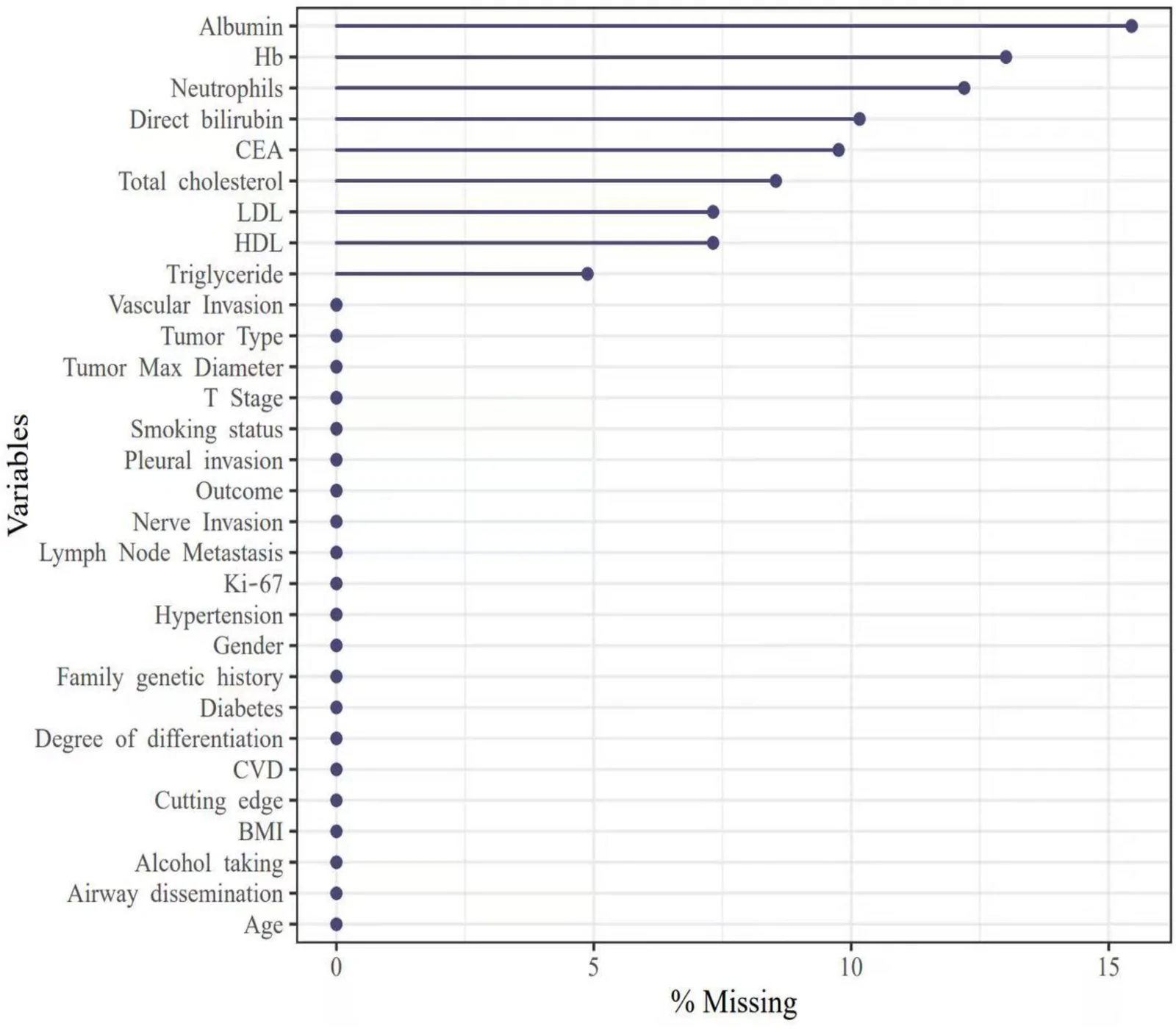
An initial missing-data audit before source-record completion. The figure summarizes missing laboratory entries identified during the first data extraction. These entries were subsequently verified against the original medical records and completed before the locked modeling analysis; no statistical imputation was used for the revised final model.

For feature selection, LASSO regression with a binomial family and L1 regularization was performed exclusively in the training set (24). Tenfold cross-validation across 1,000 candidate lambda values was used to determine the penalty parameter, and the parsimonious lambda.1se criterion was adopted. Variables retained in the training set were fixed before evaluation in the internal validation set, with no validation-set reselection.

### Machine-learning algorithms

Seven candidate algorithms were evaluated: Logistic Regression (LR), Decision Tree (DT), Random Forest (RF), Support Vector Machine (SVM), Artificial Neural Network (ANN), XGBoost, and LightGBM. Model tuning was restricted to the training set using the candidate grids specified in the Python analysis pipeline, with fivefold cross-validation used during model development. The hold-out validation set was locked until final evaluation. The final fitted settings used to generate the revised predictions were as follows: DT (ccp_alpha = 0.0, max_depth = 5, max_features = ‘sqrt’, min_samples_split = 10); RF (n_estimators = 450, max_features = 2); XGBoost (learning_rate = 0.1, max_depth = 3, n_estimators = 50, subsample = 1.0); LightGBM (colsample_bytree = 0.6, learning_rate = 0.1, n_estimators = 100, num_leaves = 31, subsample = 0.6); SVM (C = 0.1, degree = 2, gamma = ‘scale’, kernel = ‘linear’); and ANN (activation = ‘logistic’, hidden_layer_sizes = (50,)). Logistic regression was fitted without a tuning hyperparameter.

### Model evaluation

Model performance was evaluated in terms of discrimination, calibration, classification, and potential clinical net benefit. Receiver operating characteristic (ROC) curves and AUC quantified discrimination; 95% confidence intervals for the AUC were estimated by 1,000 bootstrap resamples. Calibration curves with 10 probability bins and Brier scores assessed agreement between predicted and observed risks. A decision-curve analysis evaluated the net benefit across threshold probabilities. Accuracy, precision, sensitivity, specificity, F1 score, Cohen's kappa, Youden's J, positive predictive value, and negative predictive value were calculated using a classification threshold of 0.50. Final model selection prioritized internal-validation AUC, while also considering calibration, overfitting, and the complementary performance metrics.

### Nomogram construction

A nomogram was constructed from the coefficients of the final logistic regression model fitted in the training set. The six retained predictors were represented on separate axes using the same coding applied in model development: T stage (Tis-T4), airway dissemination (no/yes), pleural invasion (no/yes), Ki-67 index (<30%/≥30%), BMI category (normal/overweight/obese), and direct bilirubin (μmol/L). Regression effects were linearly transformed to a point scale. Individual predictor points are summed to obtain total points, which are mapped to the model linear predictor and then to the estimated probability of death within 36 months through logistic transformation. The nomogram was intended as a transparent visualization of the final regression model rather than as an independently validated clinical scoring system. Because the analytic cohort was outcome-ascertained, its absolute probability scale should be regarded as model-based within this analytic framework and requires independent validation and recalibration before population-level interpretation.

### Online application

To facilitate model demonstration and exploratory use, the final logistic regression model was implemented in an interactive web-based calculator. The application uses the same six predictors and coding scheme as the locked regression model and returns a model-based estimate of death within 36 months. Because the model had undergone only internal validation and was derived from an outcome-ascertained analytic cohort, the calculator is intended only for research and model-demonstration purposes rather than direct clinical decision-making. Its absolute probabilities require external validation and recalibration before they can be interpreted as population risks.

### Statistical analysis

Statistical analyses were conducted using R version 4.4.3 for descriptive analysis and LASSO-based feature selection and Python version 3.10.4 for model development and evaluation. Source-record verification was used to complete initially missing laboratory fields; the final modeling dataset used in the revised analysis contained no missing model values, and no statistical imputation was performed. Continuous variables are presented as median (interquartile range [IQR]), unless otherwise specified, and they were compared with the Wilcoxon rank-sum test when distributional assumptions for parametric testing were not met. Categorical variables are reported as *n* (%) and were compared using the chi-square test or Fisher's exact test as appropriate. Bootstrap procedures used 1,000 resamples with a fixed random seed of 123. A two-sided *P*-value <0.05 was considered statistically significant.

All included patients had a definite 36-month outcome, and therefore, the endpoint was analyzed as a fixed-time binary classification problem rather than a time-to-event outcome. This design was selected for the available analytic dataset and does not replace survival analysis in cohorts with censoring. If patients with incomplete follow-up are retained in future studies, Cox regression, flexible survival models, or survival-based machine-learning methods should be considered so that follow-up time and censoring are modeled explicitly.

## Results

### Patient characteristics

Patient-level outcome coding and the direction of the clinicopathological variables were reaudited before the revised analysis. Outcome = 1 represented death within 36 months and Outcome = 0 represented survival beyond 36 months. Among the 350 patients with definitively ascertainable 36-month vital status, 166 (47.43%) were classified as having died within 36 months and 184 (52.57%) were alive beyond 36 months. These percentages describe the outcome composition of the analytic sample and should not be interpreted as the population-based 3-year mortality rate after pulmonary resection. The corrected baseline distributions were clinically coherent for the variables retained by LASSO. Advanced T stage was more frequent among patients who died within 36 months (T2–T4: 34.34% vs. 18.48%; *P* = 0.003). Airway dissemination (12.05% vs. 2.72%; *P* < 0.001), pleural invasion (18.07% vs. 5.98%; *P* < 0.001), and Ki-67 ≥ 30% (36.75% vs. 22.28%; *P* = 0.003) were also more frequent in the mortality group. Normal BMI was more common among deaths, whereas obesity was more common among survivors (*P* < 0.001). Direct bilirubin was higher in the mortality group [4.49 (3.68–5.49) μmol/L vs. 3.65 (2.91–4.44) μmol/L; *P* < 0.001] (Table 1).

**Table 1:** Baseline characteristics according to 36-month postoperative survival status.

Variables	Overall (*N* = 350)	Death within 36 months (*N* = 166)	Alive beyond 36 months (*N* = 184)	*P*
Gender, *n* (%)				0.989
Male	192 (54.86)	91 (54.82)	101 (54.89)	
Female	158 (45.14)	75 (45.18)	83 (45.11)	
Age, *n* (%)				0.986
18–39	10 (2.86)	5 (3.01)	5 (2.72)	
40–69	260 (74.29)	123 (74.10)	137 (74.46)	
≥70	80 (22.86)	38 (22.89)	42 (22.83)	
Tumor type, *n* (%)				0.981
Adenocarcinoma	296 (84.57)	140 (84.34)	156 (84.78)	
Squamous cell carcinoma	40 (11.43)	19 (11.45)	21 (11.41)	
Other types	14 (4.00)	7 (4.22)	7 (3.80)	
Degree of differentiation, *n* (%)				0.996
Well-differentiated	142 (40.57)	67 (40.36)	75 (40.76)	
Moderately differentiated	160 (45.71)	76 (45.78)	84 (45.65)	
Poorly differentiated	48 (13.71)	23 (13.86)	25 (13.59)	
T stage, *n* (%)				**0** **.** **003**
Tis	38 (10.86)	11 (6.63)	27 (14.67)	
T1	221 (63.14)	98 (59.04)	123 (66.85)	
T2	79 (22.57)	48 (28.92)	31 (16.85)	
T3	10 (2.86)	7 (4.22)	3 (1.63)	
T4	2 (0.57)	2 (1.20)	0 (0.00)	
Lymph-node metastasis, *n* (%)				0.994
No	312 (89.14)	148 (89.16)	164 (89.13)	
Yes	38 (10.86)	18 (10.84)	20 (10.87)	
Nerve invasion, *n* (%)				1.000
No	346 (98.86)	164 (98.80)	182 (98.91)	
Yes	4 (1.14)	2 (1.20)	2 (1.09)	
Vascular invasion, *n* (%)				0.911
No	319 (91.14)	151 (90.96)	168 (91.30)	
Yes	31 (8.86)	15 (9.04)	16 (8.70)	
Airway dissemination, *n* (%)				**<0** **.** **001**
No	325 (92.86)	146 (87.95)	179 (97.28)	
Yes	25 (7.14)	20 (12.05)	5 (2.72)	
Pleural invasion, *n* (%)				**<0** **.** **001**
No	309 (88.29)	136 (81.93)	173 (94.02)	
Yes	41 (11.71)	30 (18.07)	11 (5.98)	
Tumor max diameter, *n* (%)				0.995
≤3 cm	293 (83.71)	139 (83.73)	154 (83.70)	
>3 and ≤5 cm	48 (13.71)	23 (13.86)	25 (13.59)	
>5 and ≤7 cm	7 (2.00)	3 (1.81)	4 (2.17)	
>7 cm	2 (0.57)	1 (0.60)	1 (0.54)	
Ki-67 index, *n* (%)				**0** **.** **003**
<30%	248 (70.86)	105 (63.25)	143 (77.72)	
≥30%	102 (29.14)	61 (36.75)	41 (22.28)	
BMI, *n* (%)				**<0** **.** **001**
Normal weight	150 (42.86)	90 (54.22)	60 (32.61)	
Overweight	103 (29.43)	45 (27.11)	58 (31.52)	
Obese	97 (27.71)	31 (18.67)	66 (35.87)	
Smoking, *n* (%)				0.963
No	164 (46.86)	78 (46.99)	86 (46.74)	
Yes	186 (53.14)	88 (53.01)	98 (53.26)	
Drinking, *n* (%)				0.937
No	180 (51.43)	85 (51.20)	95 (51.63)	
Yes	170 (48.57)	81 (48.80)	89 (48.37)	
Hypertension history, *n* (%)				0.982
No	171 (48.86)	81 (48.80)	90 (48.91)	
Yes	179 (51.14)	85 (51.20)	94 (51.09)	
Diabetes history, *n* (%)				0.975
No	200 (57.14)	95 (57.23)	105 (57.07)	
Yes	150 (42.86)	71 (42.77)	79 (42.93)	
Cardiovascular disease, *n* (%)				0.921
No	193 (55.14)	92 (55.42)	101 (54.89)	
Yes	157 (44.86)	74 (44.58)	83 (45.11)	
Family genetic history, *n* (%)				0.975
No	122 (34.86)	58 (34.94)	64 (34.78)	
Yes	228 (65.14)	108 (65.06)	120 (65.22)	
HB (g/L)	131.95 (124.45–139.60)	130.90 (123.55–139.28)	132.30 (125.20–139.78)	0.305
CEA (ng/mL)	2.87 (1.61–5.07)	2.71 (1.59–5.19)	2.94 (1.61–5.01)	0.919
Neutrophils (%)	58.69 (54.12–63.48)	58.63 (54.05–63.62)	58.69 (54.15–63.25)	0.789
TC (mmol/L)	4.12 (3.61–4.64)	4.21 (3.62–4.62)	4.08 (3.56–4.65)	0.433
Albumin (g/L)	40.99 (38.80–43.21)	40.93 (38.93–43.12)	41.10 (38.73–43.26)	0.893
HDL (mmol/L)	1.17 (1.00–1.39)	1.19 (0.98–1.36)	1.16 (1.02–1.40)	0.907
LDL (mmol/L)	2.29 (1.91–2.66)	2.25 (1.84–2.60)	2.33 (1.99–2.68)	0.129
TG (mmol/L)	1.17 (0.88–1.58)	1.12 (0.87–1.51)	1.19 (0.91–1.60)	0.388
Direct bilirubin (μmol/L)	3.95 (3.13–5.00)	4.49 (3.68–5.49)	3.65 (2.91–4.44)	**<0** **.** **001**

Values are *n* (%) or median (IQR), as appropriate. Bold *P* values indicate statistically significant between-group differences (*P* < 0.05). HB, hemoglobin; TC, total cholesterol; TG, triglycerides; BMI, body mass index.

### Predictor selection

LASSO regression retained six predictors at the lambda.1se criterion: T stage, airway dissemination, pleural invasion, Ki-67 index, BMI, and direct bilirubin (Figure 3). These variables were fixed before model fitting and were used consistently across all seven candidate algorithms. Their observed directions were concordant with the corrected outcome coding: higher T stage, airway dissemination, pleural invasion, Ki-67 ≥30%, and higher direct bilirubin were associated with higher model-estimated mortality risk, whereas the BMI coding yielded lower predicted risk as BMI category increased.

**Figure 3:**
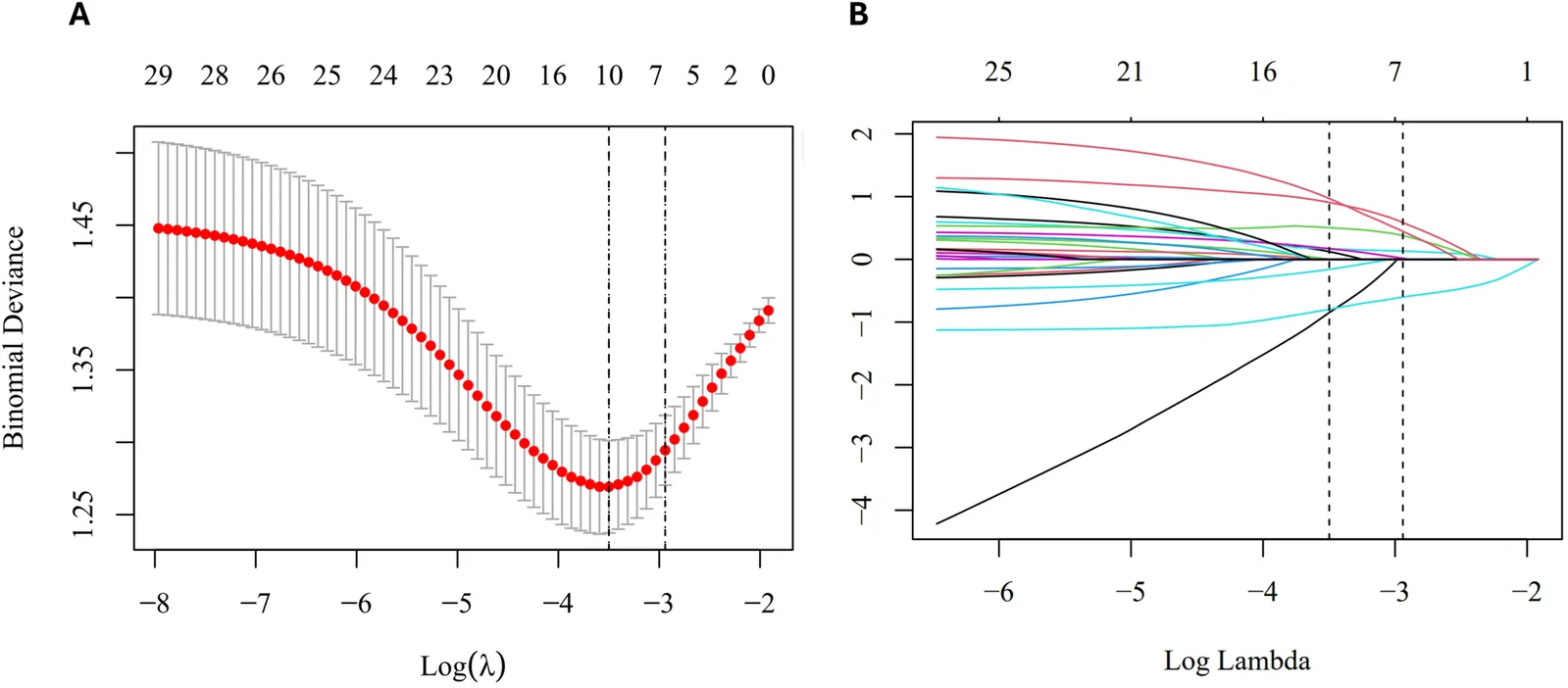
LASSO feature selection. **(A)** Ten-fold cross-validation curve showing binomial deviance across log(*λ*); the left and right dashed lines indicate *λ*min and *λ*1se, respectively. **(B)** Coefficient paths of candidate predictors across log(*λ*); the dashed lines indicate the same two values. Six predictors: T Stage, Airway dissemination, Pleural invasion, Ki-67 index, BMI, and Direct bilirubin were retained at *λ*1se.

### Multimodel integrated analysis for classification

In the training set, Random Forest showed perfect apparent discrimination and classification (AUC = 1.00; accuracy = 1.00), indicating a substantial risk of overfitting. XGBoost and LightGBM also performed strongly in training, with AUCs of 0.90 and 0.89, respectively, whereas logistic regression achieved an AUC of 0.82. Because training performance alone can be optimistic, the final model choice was based primarily on the locked internal validation set rather than on apparent fit in the development sample. Full training-set metrics are reported in Table 2.

**Table 2:** Performance metrics of machine-learning models on the training set.

Model	AUC	Accuracy	Precision	Sensitivity	Specificity	F1 score	Kappa	Youden's J	PPV	NPV
Logistic	0.82	0.76	0.76	0.71	0.80	0.73	0.51	0.51	0.76	0.75
Decision Tree	0.84	0.77	0.83	0.65	0.88	0.73	0.53	0.53	0.83	0.73
Random Forest	1.00	1.00	1.00	1.00	1.00	1.00	1.00	1.00	1.00	1.00
XGBoost	0.90	0.82	0.85	0.74	0.88	0.79	0.63	0.63	0.85	0.79
LightGBM	0.89	0.82	0.85	0.74	0.88	0.79	0.63	0.63	0.85	0.79
SVM	0.82	0.76	0.77	0.69	0.81	0.73	0.51	0.51	0.77	0.74
ANN	0.82	0.77	0.76	0.74	0.79	0.75	0.53	0.53	0.76	0.77

PPV, positive predictive value; NPV, negative predictive value.

In the internal validation set, logistic regression achieved the highest AUC at 0.79 (95% CI, 0.70–0.87), with accuracy 0.75, precision 0.74, sensitivity 0.71, specificity 0.78, F1 score 0.73, kappa 0.50, and Youden's J 0.50. The ANN and SVM showed similar discrimination (AUCs 0.78 and 0.78), whereas Random Forest decreased from an AUC of 1.00 in training to 0.73 in validation. XGBoost and LightGBM yielded validation AUCs of 0.70 and 0.71, respectively. The marked train-validation gap for Random Forest supported an overfitting interpretation. Logistic regression was therefore selected as the final model because it combined the highest validation AUC with the lowest validation Brier score (0.187) and stable overall performance (Table 3 and Figure 4).

**Table 3:** Performance metrics of machine-learning models on the internal validation set.

Model	AUC	Accuracy	Precision	Sensitivity	Specificity	F1 score	Kappa	Youden's J	PPV	NPV
Logistic	0.79	0.75	0.74	0.71	0.78	0.73	0.50	0.50	0.74	0.75
Decision Tree	0.59	0.62	0.64	0.43	0.78	0.51	0.21	0.21	0.64	0.61
Random Forest	0.73	0.68	0.66	0.67	0.69	0.67	0.36	0.36	0.66	0.70
XGBoost	0.70	0.63	0.63	0.53	0.73	0.58	0.26	0.26	0.63	0.63
LightGBM	0.71	0.62	0.62	0.53	0.71	0.57	0.24	0.24	0.62	0.63
SVM	0.78	0.72	0.72	0.67	0.76	0.69	0.44	0.44	0.72	0.72
ANN	0.78	0.76	0.75	0.73	0.78	0.74	0.52	0.52	0.75	0.77

**Figure 4:**
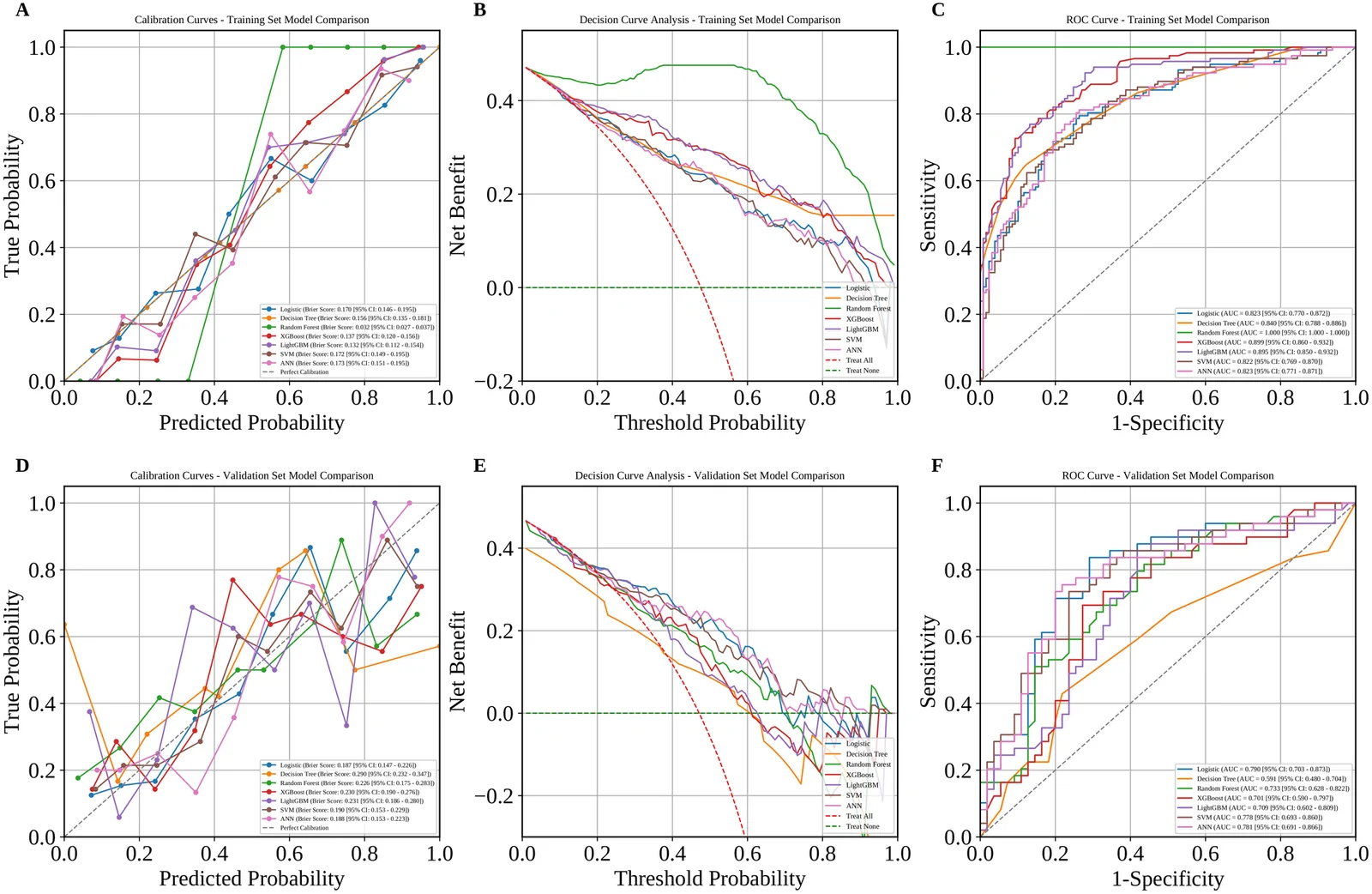
A performance comparison of seven machine-learning models. **(A)** Calibration curves in the training set. **(B)** A decision-curve analysis in the training set. **(C)** ROC curves in the training set. **(D)** Calibration curves in the internal validation set. **(E)** A decision-curve analysis in the internal validation set. **(F)** ROC curves in the internal validation set. Logistic regression achieved the highest internal-validation AUC, whereas Random Forest showed marked train-validation overfitting.

Figure 4 integrates calibration, decision-curve analysis, and ROC comparisons for the training and internal validation sets. Random Forest closely fitted the training data but lost discrimination and calibration performance in the validation set. In contrast, logistic regression retained an AUC of 0.790 (95% CI, 0.703–0.873) and a validation Brier score of 0.187 (95% CI, 0.147–0.226). Decision curve analysis (DCA) in the validation set showed that the potential net benefit varied with the decision threshold and that no model dominated across the entire threshold range. Taken together, the discrimination, calibration, and overfitting assessment supported logistic regression as the most defensible final model for subsequent visualization and exploratory implementation.

### Web-based risk calculator

The final logistic regression model was translated into a Streamlit-based online calculator using the six locked predictors. The application is publicly accessible at https://lungriskcalc.streamlit.app/. The interface allows users to enter T stage, airway dissemination, pleural invasion, Ki-67 category, BMI category, and direct bilirubin and returns the corresponding model-based estimate of 36-month mortality probability within the present analytic framework. Because outcome ascertainment may have enriched the analytic sample for mortality events, the displayed probability should not be interpreted as an externally calibrated population risk. The calculator is intended to demonstrate the fitted model and facilitate transparent risk communication and should not be used as a stand-alone treatment recommendation or substitute for clinician judgment. Independent external validation and recalibration, preferably in prospectively assembled cohorts, are required before clinical deployment. A screenshot of the updated application is shown in Figure 5.

**Figure 5:**
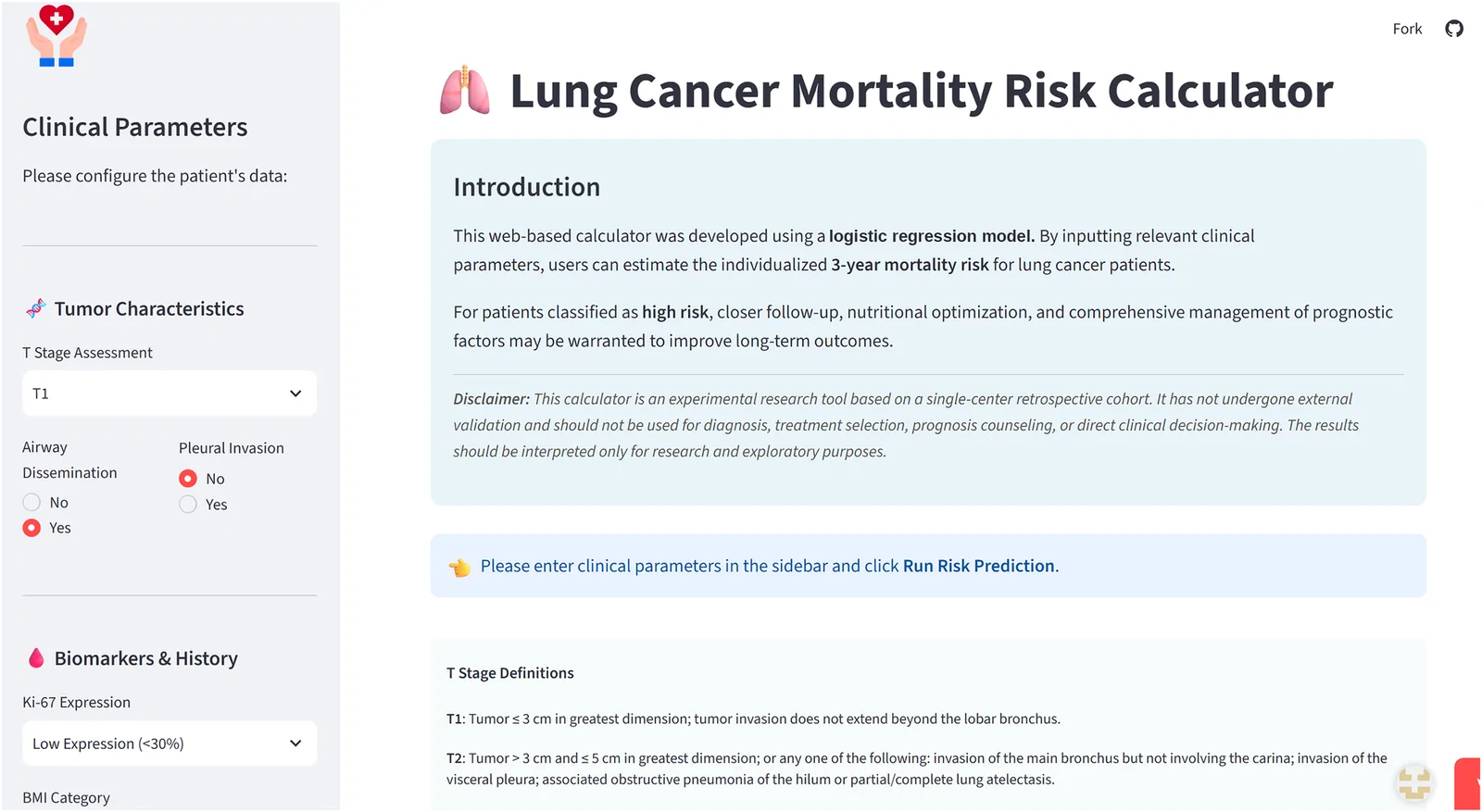
A Web-based calculator for exploratory 3-year mortality risk estimation. A Web-based calculator based on the final logistic regression model for exploratory estimation of 3-year mortality after lung cancer resection. The calculator accepts the six retained predictors and returns a model-derived probability estimate. It is intended for research and demonstration only because external and prospective validation have not yet been performed.

### Nomogram-based risk visualization

Figure 6 presents the nomogram derived from the final logistic regression model. Each of the six predictors is assigned a point value according to its model coefficient. Higher T stage, the presence of airway dissemination or pleural invasion, Ki-67 ≥30%, and increasing direct bilirubin move the patient toward higher point values and therefore a higher model-estimated probability of death within 36 months. BMI is displayed in the opposite direction because the fitted coefficient was negative under the coding normal = 1, overweight = 2, and obese = 3; consequently, normal BMI contributes more risk points than obesity. To use the nomogram, the points for all predictors are summed on the total points axis and then projected to the linear predictor and outcome scales. The figure therefore converts the regression equation into an accessible patient-level visualization, while preserving the original model structure. Because the analytic cohort may have been enriched for mortality events through outcome ascertainment, the absolute probabilities shown by the nomogram should not be interpreted as externally calibrated population risks until independent validation and recalibration are performed.

**Figure 6:**
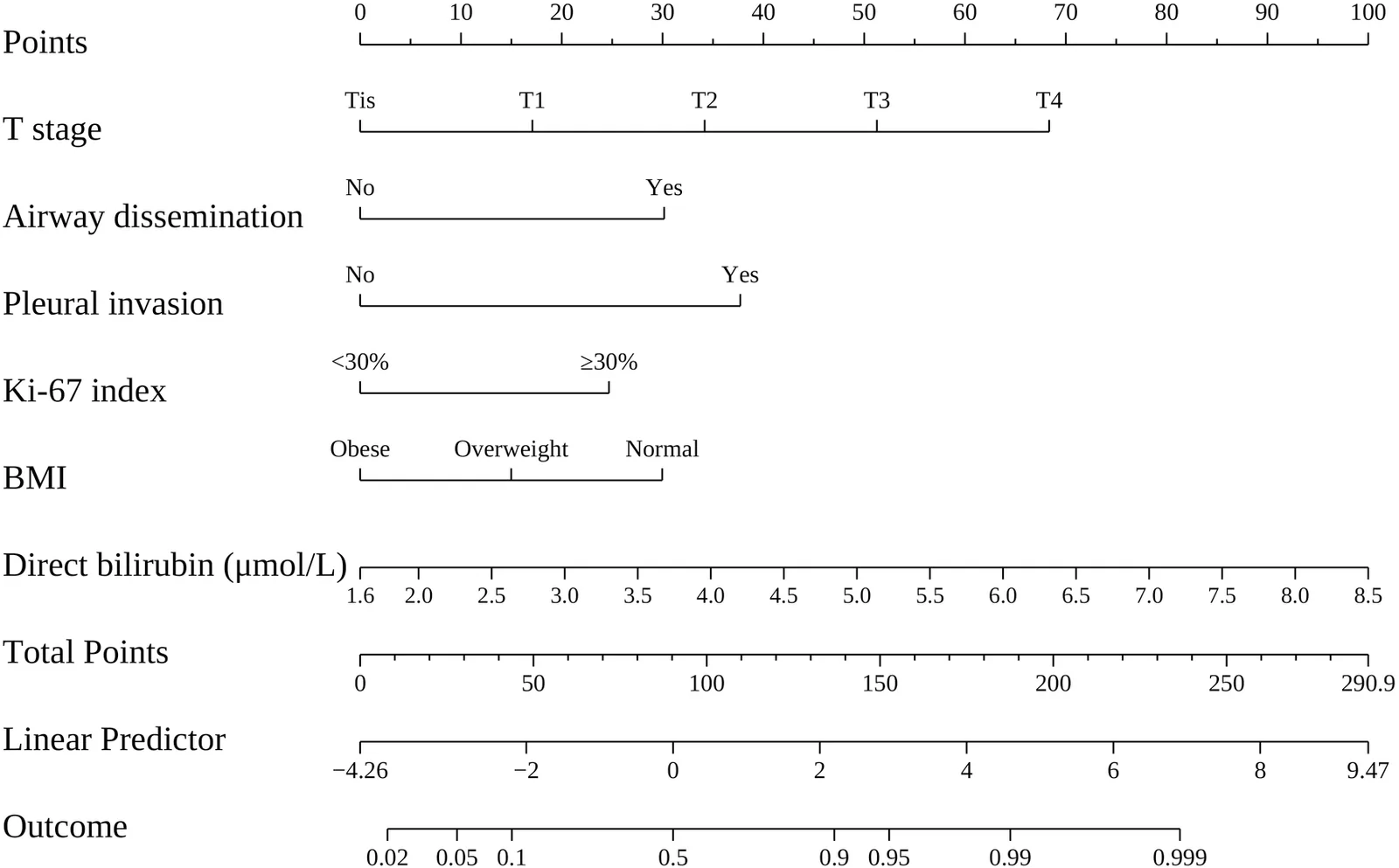
A nomogram for model-based 3-year mortality risk estimation. A nomogram derived from the final logistic regression model for model-based estimation of 3-year postoperative mortality. The nomogram includes T stage, airway dissemination, pleural invasion, Ki-67 index, BMI, and direct bilirubin. Each predictor is converted to points; the summed total points correspond to the model linear predictor and estimated outcome probability. The nomogram is a graphical representation of the internally validated regression model and is not an independently validated clinical score. Because the analytic cohort was outcome-ascertained, the displayed probabilities should not be interpreted as externally calibrated population risks before independent validation and recalibration.

## Discussion

This study developed and internally validated an exploratory model for 3-year mortality after pulmonary resection for lung cancer. Six routinely available predictors—T stage, airway dissemination, pleural invasion, Ki-67 index, BMI, and direct bilirubin—were retained by LASSO. After the patient-level outcome and category coding were reaudited, logistic regression provided the strongest overall internal validation profile, with an AUC of 0.79 and a Brier score of 0.187, and was selected as the final model. The regression model was translated into both an online calculator and a nomogram to improve transparency. These tools should be viewed as representations of the internally validated model rather than as established clinical decision–support instruments.

Previous studies on lung cancer prognosis have highlighted several clinically relevant pathological factors. For example, T stage, Ki-67 index, and pleural invasion have been reported to be associated with postoperative outcomes. A study by Wu et al. (25) emphasized the importance of T stage in lung cancer risk assessment, which is consistent with the inclusion of T stage in our final model. Similarly, Ki-67 index, a marker of cellular proliferation, has been shown to be associated with tumor aggressiveness and unfavorable outcomes in lung cancer (26, 27). Pleural invasion has also been recognized as an important pathological feature in lung cancer prognosis (28–30). In the revised analysis, higher T stage, Ki-67 ≥30%, and pleural invasion were all more frequent in the mortality group and carried risk-increasing coefficients in the final regression model. Their concordant direction supports the corrected outcome coding, although the associations should still be interpreted as model-based rather than causal effects.

Interestingly, BMI and direct bilirubin were also retained by feature selection and contributed to the final model. BMI may reflect nutritional reserve, metabolic status, or systemic condition, which have been reported to influence cancer outcomes. Previous studies have described the so-called obesity paradox in lung cancer and other malignancies, suggesting that higher BMI may sometimes be associated with more favorable outcomes, possibly through better nutritional status or treatment tolerance (31, 32). Direct bilirubin may reflect hepatic metabolism, systemic inflammation, or underlying physiological status. Previous studies have reported associations between bilirubin-related indicators and outcomes in lung cancer patients (33–35). In the revised model, higher BMI category carried a negative coefficient, whereas direct bilirubin carried a positive coefficient and was higher in the mortality group; these findings should be interpreted as model-contributing associations rather than independent causal prognostic effects.

The revised multimodel comparison also changes the interpretation of algorithm performance. Random Forest achieved an AUC of 1.00 in the training set but only 0.73 in internal validation, a pattern strongly suggestive of overfitting. Logistic regression, by contrast, showed a smaller train-validation difference (0.82 vs. 0.79) and the lowest validation Brier score among the seven candidates. The choice of the final model therefore reflects generalization and calibration rather than training-set fit. The resulting nomogram further improves interpretability because the direction and relative contribution of each retained predictor can be inspected directly without relying on a *post hoc* explanation layer.

Several limitations require explicit consideration. First, the study was retrospective and single-center, and the validation cohort was an internal hold-out subset rather than an independent external sample. Second, the fixed 36-month endpoint required a definitive ascertainment of vital status at 36 months. This cohort-construction strategy could have preferentially retained early deaths because death occurring within 36 months immediately established the endpoint, whereas patients remaining alive required a sufficiently long follow-up to establish non-event status. Consequently, patients who were alive but whose available follow-up had not yet reached 36 months could not contribute to the analytic cohort. This asymmetric ascertainment mechanism may have enriched the analytic sample for mortality events and therefore provides a plausible explanation for the relatively high event proportion of 47.43%. Importantly, this event fraction should not be interpreted as an estimate of the population-level 3-year mortality after lung cancer resection. Because the historical screening log was not sufficiently detailed to reliably reconstruct the exact number excluded solely for inadequate follow-up, the magnitude of this selection effect could not be quantified retrospectively. We therefore did not infer or estimate an unsupported exclusion count. Future studies should prospectively define follow-up windows and use time-to-event methods that retain censored observations. Third, the perfect training performance of Random Forest illustrates model instability and overfitting risk in this sample size. Fourth, complete tumor-node-metastasis (TNM) stage, detailed N and M stage, Eastern Cooperative Oncology Group (ECOG) performance status, treatment modality, molecular alterations, and recurrence information were not consistently available and could not be incorporated. Finally, the nomogram and online calculator are derived from the same internally validated logistic regression model; because absolute risk calibration may be affected by the outcome-ascertained cohort composition, their probability estimates require independent multicenter validation and recalibration before clinical use.

## Conclusion

Logistic regression showed the most defensible internal validation performance for classifying 36-month mortality status after lung cancer resection and was selected as the final model. The model was presented as an online calculator and nomogram for transparent, exploratory risk estimation. Because the analytic cohort was restricted to patients with definitively ascertainable 36-month status, the observed 47.43% event fraction reflects the composition of this outcome-ascertained sample rather than a population-level mortality estimate, and the model's absolute probabilities should not be assumed to transport directly to unselected surgical populations. Independent multicenter and prospective validation with time-to-event analysis and recalibration is required.

## Data Availability

The patient-level datasets are not publicly available because they contain sensitive clinical information and are subject to institutional and ethical restrictions. De-identified data may be made available by the corresponding author upon reasonable request, subject to approval by Tongling People's Hospital, applicable ethics requirements, and a data-use agreement. The Python scripts used for model fitting, internal validation, ROC analysis, calibration analysis, decision curve analysis, and generation of the nomogram are provided as Supplementary Code S1. Exact rerunning of the analysis requires the de-identified analytic input data, which may be made available upon reasonable request subject to institutional approval, applicable ethical requirements, and a data-use agreement. The code package contains no identifiable or patient-level clinical data.
